# Added value of ^18^F-FDG-PET/CT and cardiac CTA in suspected transcatheter aortic valve endocarditis

**DOI:** 10.1007/s12350-019-01963-x

**Published:** 2019-12-02

**Authors:** Ali R. Wahadat, Wilco Tanis, Laurens E. Swart, Asbjørn Scholtens, Gabriel P. Krestin, Nicolas M. D. A. van Mieghem, Carolina A. M. Schurink, Tycho I. G. van der Spoel, Floris S. van den Brink, Tessel Vossenberg, Riemer H. J. A. Slart, Andor W. J. M. Glaudemans, Jolien W. Roos-Hesselink, Ricardo P. J. Budde

**Affiliations:** 1 Department of Radiology and Nuclear Medicine, Erasmus Medical Center, Rotterdam, The Netherlands; 2 Department of Cardiology, Thoraxcenter, Erasmus Medical Center, Rotterdam, The Netherlands; 3 Department of Cardiology, Haga Teaching Hospital, The Hague, The Netherlands; 4 Department of Nuclear Medicine, Meander Medical Center, Amersfoort, The Netherlands; 5 Department of Medical Microbiology and Infectious Diseases, Erasmus Medical Center, Rotterdam, The Netherlands; 6 Department of Internal Medicine, Erasmus Medical Center, Rotterdam, The Netherlands; 7 Department of Cardiology, Utrecht Medical Center, Utrecht, The Netherlands; 8 Department of Cardiology, St. Antonius Hospital, Nieuwegein, The Netherlands; 9 Department of Cardiology, Medical Center Leeuwarden, Leeuwarden, The Netherlands; 10 Department of Nuclear Medicine & Molecular Imaging, Medical Imaging Center, University Medical Center of Groningen, Groningen, The Netherlands; 11 Departments Radiology, Erasmus MC, NA-2618, Dr. Molewaterplein 40, 3015GD Rotterdam, The Netherlands

**Keywords:** Infection, valvular heart disease, CT, PET

## Abstract

**Backgrounds:**

Transcatheter-implanted aortic valve infective endocarditis (TAVI-IE) is difficult to diagnose when relying on the Duke Criteria. Our aim was to assess the additional diagnostic value of ^18^F-fluorodeoxyglucose (^18^F-FDG) positron emission/computed tomography (PET/CT) and cardiac computed tomography angiography (CTA) in suspected TAVI-IE.

**Methods:**

A multicenter retrospective analysis was performed in all patients who underwent ^18^F-FDG-PET/CT and/or CTA with suspected TAVI-IE. Patients were first classified with Duke Criteria and after adding ^18^F-FDG-PET/CT and CTA, they were classified with European Society of Cardiology (ESC) criteria. The final diagnosis was determined by our Endocarditis Team based on ESC guideline recommendations.

**Results:**

Thirty patients with suspected TAVI-IE were included. ^18^F-FDG-PET/CT was performed in all patients and Cardiac CTA in 14/30. Using the Modified Duke Criteria, patients were classified as 3% rejected (1/30), 73% possible (22/30), and 23% definite (7/30) TAVI-IE. Adding ^18^F-FDG-PET/CT and CTA supported the reclassification of 10 of the 22 possible cases as “definite TAVI-IE” (5/22) or “rejected TAVI-IE” (5/22). This changed the final diagnosis to 20% rejected (6/30), 40% possible (12/30), and 40% definite (12/30) TAVI-IE.

**Conclusions:**

Addition of ^18^F-FDG-PET/CT and/or CTA changed the final diagnosis in 33% of patients and proved to be a valuable diagnostic tool in patients with suspected TAVI-IE.

**Electronic supplementary material:**

The online version of this article (10.1007/s12350-019-01963-x) contains supplementary material, which is available to authorized users.

## Introduction

Transcatheter aortic valve implantation (TAVI) is now an accepted and widely applied treatment for aortic valve stenosis in selected patient populations.^1^ As a major complication, prosthetic heart valve endocarditis (PVE) after a TAVI (TAVI-IE) has been reported to occur with an incidence of 1.6 events per 100 person-years.^2^ However, the timely diagnosis of this serious disease remains a challenge when using only the modified Duke Criteria because transthoracic or transesophageal echocardiography (TTE and TEE) may be impaired by artifacts (acoustic shadowing/reverberation) caused by the metallic stent around the valve.

The most recent European Society of Cardiology (ESC) guidelines for infectious endocarditis introduced ^18^F-fluorodeoxyglucose (^18^F-FDG) positron emission/computed tomography (PET/CT) and cardiac computed tomography angiography (CTA) as additional diagnostic tools for suspected PVE.^3^ For surgically implanted prosthetic valves, several reports have described the additional value of ^18^F-FDG-PET/CT (both visual and quantitative assessment) and CTA in diagnosing PVE as well as how to acquire and interpret the images.^4^–^8^ In suspected TAVI-IE, these additional imaging tools also may have diagnostic value resulting in a different treatment strategy; however, reports on TAVI-IE are still very scarce.^9^

The purpose of this study was to assess the additional diagnostic value of ^18^F-FDG-PET/CT and/or cardiac CTA in patients suspected of TAVI-IE when added to the modified Duke Criteria.

## Materials and Methods

### Patient Selection

All patients with a history of TAVI who were referred to six different hospitals and underwent either ^18^F-FDG-PET/CT and/or cardiac CTA for suspicion of TAVI-IE were retrospectively included in this study. The institutional medical ethics committee approved the study and waived the need for informed consent.

### Patient Classification

All data were extracted from the electronic patient records in each hospital. Both the modified Duke Criteria (echocardiographic findings, blood cultures, and clinical features) and the 2015 ESC criteria (modified Duke Criteria with the addition of ^18^F-FDG-PET/CT and CTA) were used to score each patient and give them interim diagnoses.^3^ The final diagnosis (either rejected, possible, or definite TAVI-IE) was established by consensus via the multidisciplinary Endocarditis Team in each hospital, using the latest ESC criteria and all clinical records. This meeting was scheduled within 1 to 7 days after all clinical data (including PET/CT and the eventual CTA) were available. Participants of this multidisciplinary meeting included at least a cardiologist, cardiothoracic surgeon, an infectious disease specialist, and a cardiac radiologist/nuclear medicine physician.

### Blood Cultures

Blood culture results from the period in which patients were hospitalized were included and used for analysis. Blood cultures were deemed positive according to the modified criteria in the latest ESC guidelines for infective Endocarditis.^3^

### Echocardiography

Either TTE, TEE, or both were performed in all included patients, following the current guidelines. The examinations were reported by a certified cardiologist as part of clinical practice and the clinical reports were used for this study. TTE/TEE was considered positive if at least one echo demonstrated the presence of an anatomical and/or echocardiographic criteria for endocarditis according to the ESC guidelines.^3^.

### Image Acquisition

#### ^18^F-Fdg-Pet/Ct

Patients followed a 24-hour low carbohydrate diet (of which the last 12 hours were spent fasting) to induce free fatty acid metabolism and suppress glucose metabolism in the myocardium.^10^–^12^ One hour after an intravenous ^18^F-FDG injection [on average 215 megabecquerel (MBq)], a total body or skull-midthigh ^18^F-FDG-PET/CT scan was acquired using a Siemens Biograph mCT/mCT flow or Philips Gemini TF camera system. Additionally, a low dose CT was performed for attenuation correction.

#### CT angiography

CTA imaging was performed on a dual source CT scanner (Siemens, SOMATOM FORCE or Flash). Scans were performed either with retrospective ECG-gating or a dedicated CT acquisition protocol with ECG-gating tailored to the imaging of prosthetic heart valves to provide optimal image quality at minimal radiation exposure.^13^

### Image Analysis and Interpretation

#### PET analysis

Visual analyses of ^18^F-FDG-PET/CT images had been performed by a nuclear medicine physician as part of clinical practice, while additional quantitative ^18^F-FDG-PET/CT analyses were performed by an experienced nuclear medicine physician (AS, RS).

The maximum standardized uptake value (SUV_max_) was measured in an automated volume of interest (VOI) with a 40% isocontour around the valve on reconstructions that were provided through a standardized calibration and reconstruction method by the European Association of Nuclear Medicine Research Ltd (EARL) when available.^7^ The target to background ratio (SUV_ratio_) was then calculated as the ratio of the SUV_max_ of the valve and the SUV_mean_ of the blood pool in the descending aorta, not including the vessel wall. In all available cases, these measurements were also performed in non-EARL accredited reconstructions.

Additionally, extra cardiac ^18^F-FDG uptake was defined as either physiological, possible embolization, pathological lymph node, or extra cardiac infections/inflammation.

#### Cardiac CTA analysis

The CTA scans had been reported by a cardiac radiologist as part of clinical practice. We used the original clinical report to score for signs of infectious endocarditis (vegetations, mycotic aneurysms, abscesses, paravalvular leakage, and valve dehiscence).

### Statistics

For analysis of our main outcomes, descriptive statistics was used. Non-parametric statistical analyses (Mann-Whitney U test) were performed for the comparison of continuous variables in rejected and definite TAVI-IE. The interquartile ranges (IQR) and confidence intervals (CI) were denoted in square brackets. A significance level of *P* = 0.05 and 95% CIs were used. In case of missing data, patients were excluded from analyses of certain parameters. SPSS statistics v24.0 (IBM Corp) was used for all analyses.

### Follow-Up

Information on patient follow-up was derived from the electronic patient records in each hospital. Follow-up time was defined as the period between the admission date until the date of the last notation in the clinical records. Data about mortality were derived from the Central Bureau for Statistics (CBS) database (100% available).

## Results

### Patient Characteristics and Classification

**Figure 1:**
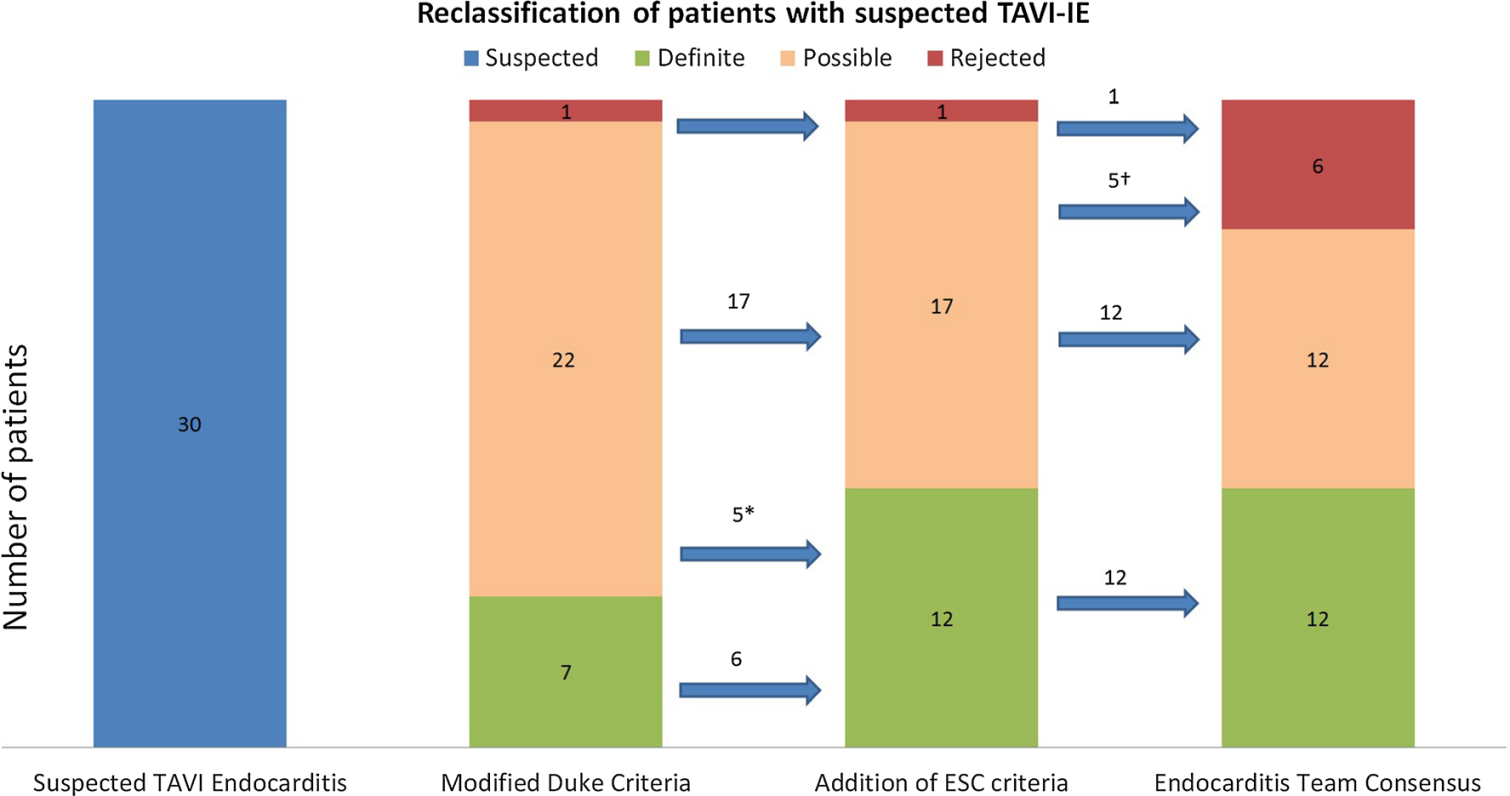
Distribution of patients with suspected Endocarditis based on Modified Duke Criteria, ESC criteria, and Endocarditis Team consensus based on ESC criteria

**Figure 2:**
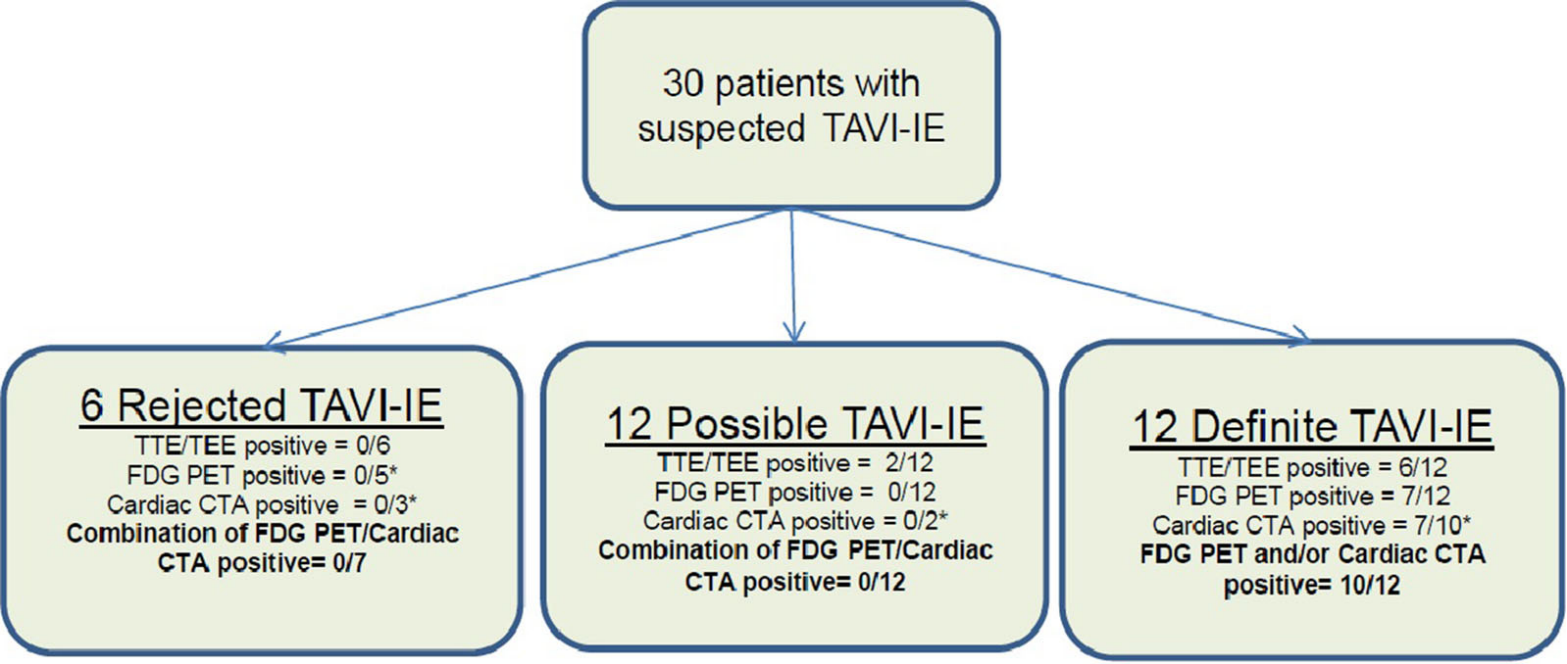
Positive results of either TTE/TEE, FDG-PET, and Cardiac CTA in each group with final diagnosis of rejected, possible, and definite TAVI-IE

In total, 30 patients (mean age ± SD 77 ± 11; 17 males) with an initial suspicion of TAVI-IE were identified and included in this study. Valve types included 15 Corevalves, 8 Edwards Sapien, and 7 others that, on average, had been implanted 278 days [104 to 768] (median with IQR) before ^18^F-FDG-PET/CT imaging. Baseline patient characteristics are detailed in Table 1. A detailed overview of all results per patient is given in Online Resource 1. Based on the modified Duke criteria, 7/30 patients (23%) had the diagnosis of “definite TAVI-IE”, 22/30 patients (73%) “possible TAVI-IE”, and 1/30 patients (3%) “rejected TAVI-IE”. After addition of ^18^F-FDG-PET/CT and/or CTA, 12/30 patients (40%) had a final diagnosis of “definite TAVI-IE” based on Endocarditis Team consensus, whereas in 6/30 patients (20%) the diagnosis of endocarditis was rejected after additional diagnostic workup. In the remaining 12/30 patients (40%), the diagnosis of “possible TAVI-IE” was concluded. These patients were assigned and treated as “definite TAVI-IE”. Overall 10 patients (33%) were reclassified as detailed in Figure 1. None of the patients underwent surgery. During a median follow-up of 481 [116 to 1060] days mortality was observed in 14/30 patients, including 6/12 patients with definite endocarditis, 4/12 with possible, and 4/6 with rejected endocarditis (Figure 2).

**Table 1:** Patient characteristics

	All patients with suspicion of TAVI endocarditis	Definite TAVI endocarditis	Possible TAVI Endocarditis	Rejected TAVI endocarditis (after initial suspicion)
Demographics	n = 30	n = 12	n = 12	n = 6
Age, mean ± SD, years	77 ± 11	73 ± 9	79 ± 12	79 ± 11
Gender, male, n (%)	17 (57)	6 (50)	7 (58)	4 (67)
BMI median [IQR], kg/m^2^	26 [23–32]	26 [21–31]	25 [23–30]	29 [23–34]
Prior history of endocarditis, n (%)	0 (0)	0 (0)	1 (8)	0 (0)
Time since valve implantation, median [IQR], days	278 [104–768]	116 [60–699]	632 [219–1451]	125 [104–462]
Valves implanted < 3 months prior to PET, n (%)	6 (20)	4 (13)	1 (3)	1 (3)
Type of valve, n (%)				
Corevalve	15 (50)	5 (42)	5 (42)	5 (83)
Sapien	8 (29)	2 (17)	5 (42)	1 (17)
Lotus	4 (14)	4 (33)	0 (0)	0 (0)
Portico	1 (4)	0 (0)	1 (8)	0 (0)
Directflow	2 (8)	1 (8)	1 (8)	0 (0)
Valve in valve TAVI, n (%)	0 (0)	0 (0)	0 (0)	0 (0)
Device, n (%)				
1 lead pacemaker	2 (7)	0 (0)	1 (8)	1 (17)
2 lead pacemaker	6 (20)	2 (17)	1 (8)	3 (50)
ICD/CRT-P/CRT-D	0 (0)	0 (0)	0 (0)	0 (0)
Bloodcultures available, n (%)	30 (100)	12 (100)	12 (100)	6 (100)
Positive blood cultures, n (%)				
E. faecalis	12 (40)	4 (33)	5 (42)	3 (50)
Streptococci	8 (27)	3 (25)	5 (42)	0 (0)
S. aureus	2 (7)	2 (17)	0 (0)	0 (0)
S. lugudensis	2 (7)	1 (8)	0 (0)	1 (17)
S. epidermidis	2 (7)	1 (8)	0 (0)	1 (17)
Mycobacterium	1 (3)	0 (0)	0 (0)	1 (17)
Abscessus	1 (3)	0 (0)	1 (8)	0 (0)
Lactobacillus rhamnosus	2 (7)	1 (8)	1 (8)	0 (0)
Negative blood cultures				
Days of IV antibiotic therapy prior to ^18^F-FDG-PET/CT, median[IQR]	9 [7–14]	10 [7–14]	8 [6–14]	11 [7–25]
CRP^a,b^, median[IQR], mg/L	47 [15–106]	35 [10–57]	86 [26–149]	28 [8–145]
Leukocytes^a,b^, median[IQR], ×109/L	8.5 [6.3–11.7]	7.5 [6.3–11.7]	10.3 [7.6–13.9]	5.5 [5.0–8.7]
Median follow-up period[IQR] (days)^c^	481 [116–1060]	760 [119–1140]	793 [149–1139]	123 [91–252]
All-cause mortality, n (%)	14 (47)	6 (50)	4 (33)	4 (67)

^a^CRP and leucocytes levels on the day closest to the ^18^F-FDG-PET/CT date were selected

^b^In one patient the level of CRP and in 2 patients the level of CRP and Leucocytes prior to the ^18^F-FDG-PET/CT scan were missing. These patients were excluded from analyses

^c^The numbers were derived from the most recent notes in the electronic patient files

### Blood Cultures

Blood culture results were available for all patients and were positive at least once in 29/30 patients. *Enterococcus faecalis* was the most common type of microorganism in patients with a final diagnosis of “definite TAVI-IE” (4/12) and those with “rejected TAVI-IE” (3/6).

### Echocardiography

The reports of TTE and/or TEE were available in all cases. TTE and/or TEE was positive in 6/12 patients with a final diagnosis of “definite TAVI-IE” and in 2/12 patients with “possible TAVI-IE” (1 with negative blood cultures and 1 with positive blood cultures but not meeting the major ESC criteria). In the “rejected TAVI-IE” group TTE and TEE were negative in all cases.

### ^18^F-FDG-Pet/CT

#### Visual analysis

^18^F-FDG-PET/CT was performed in all patients. All scans were available for further quantitative analyses except one which could not be analyzed quantitatively due to technical difficulties. The “time since implantation”, “the days of antibiotic therapy prior to the scan,” and “serum levels of CRP and leucocytes” were not significantly different between positive-and negative-reported ^18^F-FDG-PET/CT scans (Table 2).

**Table 2:** Time interval from implantation, infection parameters, days of iv antibiotic therapy, SUV_max_ , and SUV_ratio_ around the prosthetic valve prior to ^18^F-FDG-PET/CT in patients with a positive-reported and negative-reported ^18^F-FDG-PET/CT scan

	Positive-reported ^18^F-FDG-PET/CT	Negative-reported ^18^F-FDG-PET/CT
Time since valve implantation, median [IQR], days	126 [76–557]	393 [105–1212], *P* = 0.29*
CRP, median [IQR], mg/L	25 [11–53]	62 [18–127], *P =* 0.15*
Leukocytes, median [IQR], ×109/L	8.0 [7.0–11.0]	9.6 [6.0–12.5], *P =* 0.63*
Days of IV antibiotic therapy prior to ^18^F-FDG-PET/CT, median [IQR]	10 [9–14]	9 [7–14], *P =* 0.48*
SUV_max_, median [IQR]	5.5 [3.8–7.1]	3.6 [3.4–4.4], *P =* 0.01*
SUV_ratio_, median [IQR]	2.9 [2.0–3.7]	1.9 [1.7–2.1], *P =* 0.04*

*Comparison between positive-reported and negative-reported ^18^F-FDG-PET/CT groups

**Figure 3:**
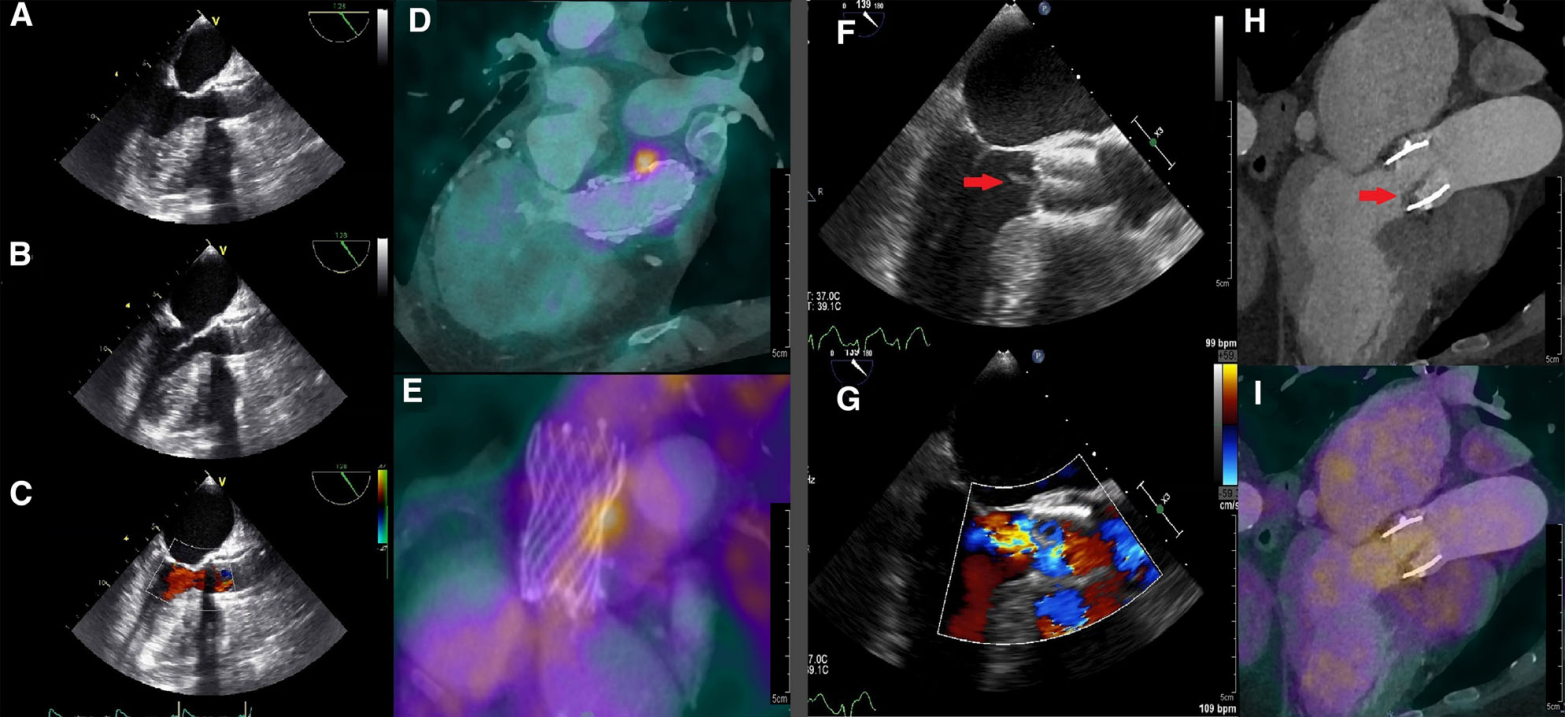
Two cases of one positive PET/CT and one negative PET/CT for TAVI-IE. Case 1 (**A** to **E**): A 75-year-old female with suspected Corevalve TAVI-IE who underwent a TEE without signs of endocarditis (**A** to **C**). PET/CT images (**D**/**E**) demonstrated focal FDG uptake alongside the corevalve as positive sign of TAVI-IE. This case was previously published as a case report.^14^ Case 2 (**F** to **I**): An 81-year-old female with suspected Edwards-Sapien TAVI-IE who underwent a TEE (**F**/**G**) with a vegetation on the aortic valve and mild aortic regurgitation. CTA demonstrated thickening of the aortic valve leaflets (**H**) as possible signs of vegetation. However, PET/CT images (**I**) showed no focal ^18^F-FDG uptake on the leaflets. This was explained by the low inflammatory activity and 2 weeks of intravenous antibiotic therapy prior to the PET/CT scan

^18^F-FDG-PET/CT was reported positive in 7 patients who all had a diagnosis of “definite TAVI-IE” (58%) (Figure 3A–E). In all cases of “possible TAVI-IE” (n = 12) and “rejected TAVI-IE” (n = 6), ^18^F-FDG-PET/CT was reported as negative. Additionally, a negative ^18^F-FDG-PET/CT report was given in 5/12 patients with “definite TAVI-IE” (42%), including 2 with very low CRP levels (< 10 mg/L); 2 with moderate cardiac suppression due to high serum glucose levels during the scan (> 10 mmol/L); and 1 with no signs of endocarditis on any of the imaging modalities, but a final diagnosis of definite TAVI-IE (positive blood cultures, prosthetic heart valve, fever, and cerebral embolization).

Extra cardiac ^18^F-FDG uptake was noticed in 19 patients, including 9 patients with a final diagnosis of definite TAVI-IE. Five patients were reclassified as rejected TAVI-IE after the ^18^F-FDG-PET/CT demonstrated abnormal ^18^F-FDG uptake elsewhere in the body, indicating an alternative infection that explained the clinical symptoms (without any signs of it being a septic embolic complication of endocarditis).

#### Quantitative analysis

EARL-reconstruction images were available in 20/30 (67%) cases and non-EARL-reconstruction images in 29/30 patients for further quantitative analyses. For both EARL and non-EARL standardized scans, the SUV_max_ and SUV_ratio_ did not differ significantly between patients with definite TAVI-IE and rejected TAVI-IE. These SUV measurements are described in detail in Table 3.

**Table 3:** SUV_max_ and SUV_ratio_ on the ^18^F-FDG-PET/CT scans for patients with definite, possible, and rejected TAVI-IE

All EARL standardized scans	Definite TAVI-IE	Possible TAVI-IE	Rejected TAVI-IE
	n = 8	n = 7	n = 5
SUV_max_, median [IQR]	3.6 [2.8–4.8]	3.3 [3.1–3.8]	3.6 [3.3–3.9] *P* = 0.83*
SUV_ratio_, median [IQR]	2.0 [1.7–2.2]	1.9 [1.5–2.1]	1.7 [1.3–2.3] *P* = 0.38*

Non-EARL standardized scans	Definite TAVI-IE	Possible TAVI-IE	Rejected TAVI-IE
	n = 12	n = 11	n = 6
SUV_max_, median [IQR]	4.1 [3.5–5.8]	3.5 [3.2–3.8]	4.2 [3.4–4.5] *P* = 0.85*
SUV_ratio_, median [IQR]	2.3 [1.7–2.9]	2.0 [1.7–2.1]	1.9 [1.8–2.3] *P* = 0.40*

*Comparison of “definite TAVI-IE” and “rejected TAVI-IE” groups

There was a significant difference between the SUV_max_ and SUV_ratio_ measured in the positive-reported ^18^F-FDG-PET/CT scans compared to the negative-reported ^18^F-FDG-PET/CT scans.

### CT Angiography

Cardiac CTA was performed in 14/30 patients (47%) including 9/12 patients with definite, 2/12 with possible, and 3/6 with rejected TAVI-IE. Positive signs of endocarditis such as vegetation (n =5), mycotic aneurysm (n = 1), and both vegetation and mycotic aneurysm (n = 1) were noticed in 7/9 (78%) patients with “definite TAVI-IE” (CTA not performed in 3/12 patients with definite endocarditis). The other 2/9 patients with definite TAVI-IE but negative CTA either had positive signs of TAVI-IE on the ^18^F-FDG-PET/CT (1/2) or TTE/TEE (1/2). Three out of 7 patients with a positive CTA had no signs of endocarditis on the TTE/TEE. The mycotic aneurysms detected in 2 cases on CTA were not visible on TTE/TEE

### Impact of ^18^F-FDG-PET/CT and CTA

^18^F-FDG-PET/CT helped to reclassify 8 patients from the initial possible TAVI-IE group to either the definite TAVI-IE group (3/8) or the rejected TAVI-IE group (5/8). Additionally, CTA aided in the reclassification of an additional 2 patients that had a normal ^18^F-FDG-PET/CT by identifying vegetations or other structural abnormalities, while strengthening the reclassification by ^18^F-FDG-PET/CT in 4 patients by also depicting structural abnormalities when increased ^18^F-FDG uptake had already been identified. Details of reclassification and the number of imaging techniques used in each group are demonstrated in Figures 1 and 2.

## Discussion

In daily clinical practice, patients with a prosthetic valve who show signs of unexplained infection and develop positive blood cultures are highly suspected for endocarditis. Even if echocardiography does not show any signs of endocarditis, these patients may be pragmatically treated as such, however, this has major clinical implications. If ^18^F-FDG-PET/CT shows signs of infection elsewhere without any signs of endocarditis, this may lead to a change in diagnosis and reduction of the antibiotic treatment period. On the other hand, if the diagnosis is changed to definite endocarditis due to ^18^F-FDG-PET/CT findings, the antibiotic treatment may be prolonged or even adjusted to lifelong suppression therapy. All these changes might have effects on morbidity and mortality.

**Figure 4:**
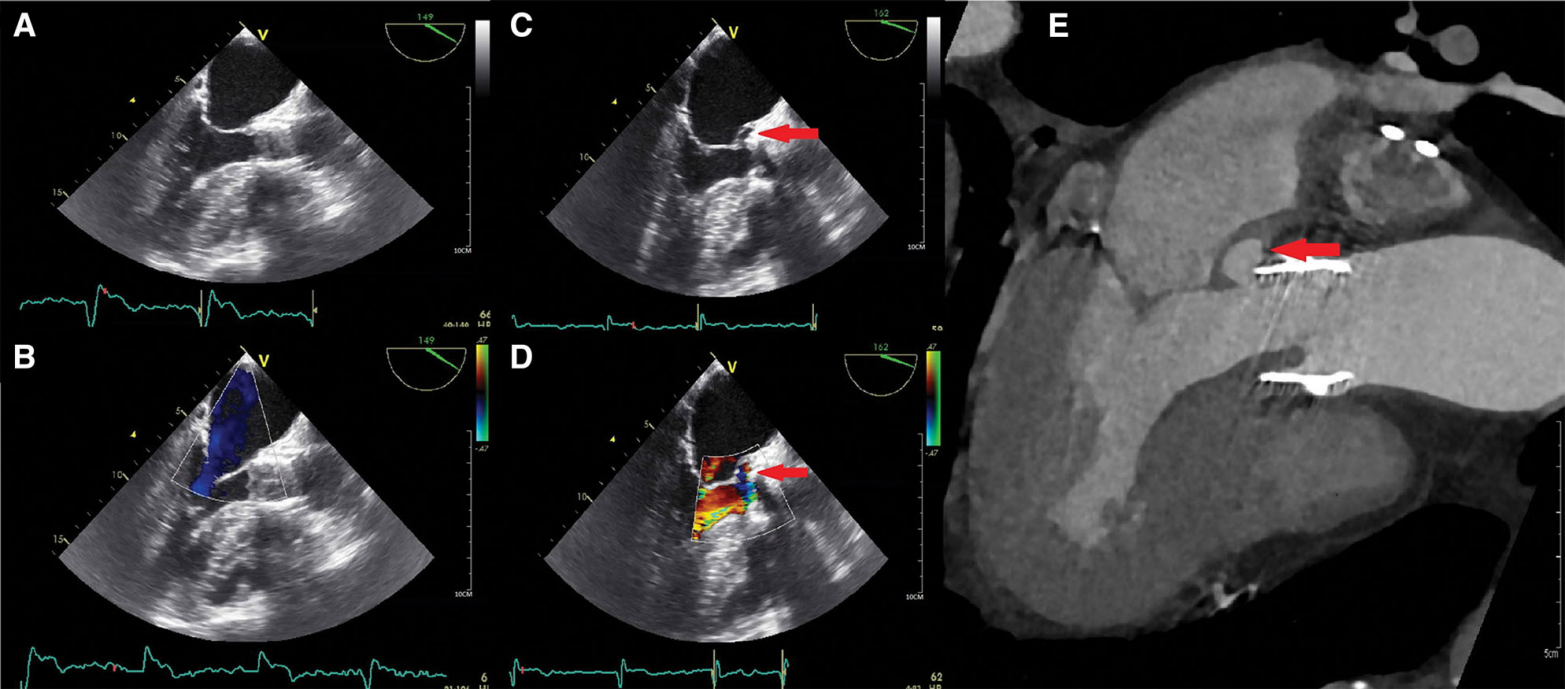
CTA images of a 77-year-old male with suspected TAVI-IE. Initial TEE (**A**, **B**) showed only thickened aortic valve leaflets as signs of vegetation. Repeating TEE after a few days (**C**, **D**) showed a new aortic regurgitation and a paravalvular space as sign of possible mycotic aneurysm, which was confirmed on the CTA (**E**)

Our study showed that the use of ^18^F-FDG-PET/CT and/or CTA resulted in reclassification of 10/22 (45%) patients with an initial diagnosis of “possible TAVI-IE”. Furthermore, the addition of ^18^F-FDG-PET/CT led to alternative diagnoses in 4 patients initially suspected of TAVI-IE. CTA was not performed in all patients (14/30, 47%), but was positive for signs of TAVI-IE in a substantial number of patients with the final diagnosis of “definite TAVI-IE” (7/9; 78%) by demonstrating vegetations and/or mycotic aneurysms that were not seen on TTE/TEE (4/9; 44%) (Figure 4).

Although we did not encounter them in this study, false positive ^18^F-FDG-PET/CT results can occur in PVE and therefore cautious interpretation of ^18^F-FDG-PET/CT scans is advised, particularly taking into account the known confounders.^7^,^12^ Potentially, chronic inflammation and thus a false ^18^F-FDG uptake might also be caused by continuous movement and friction of the transcatheter-implanted valve. Moreover, the presence of calcifications on the native aortic valve, which are not removed during a TAVI procedure, may cause artifacts and thus false positive ^18^F-FDG-PET/CT results. Overcorrection of the ^18^F-FDG uptake signal inside the valve ring may occur during the attenuation correction (AC) due to (artifacts coming from) the metal stent around the TAVI prosthesis, necessitating side-by-side interpretation of AC and non-AC images. In a recent large study of patients suspected of PVE (including TAVI-IE), recent valve implantation was not found to be a significant predictor of a false positive ^18^F-FDG-PET/CT scan.^6^ In addition, the inflammation response caused by percutaneously implanted valves may even be less compared to the surgically implanted valves.

False negative ^18^F-FDG-PET/CT results can occur due to negative confounding effects such as low inflammatory activity caused by antibiotic treatment before the ^18^F-FDG-PET/CT^6^,^14^ (Figure 3F–I).

The standardization of calibration and reconstruction method between centers remains challenging and EARL reconstruction is not formally recommended for cardiac purposes. In our study we performed the quantitative analysis on both the EARL- as well as the non-EARL-reconstruction images and on both analyses we did not find a statistically significant difference between the rejected TAVI-IE and the definite TAVI-IE groups.

In a recent study, quantitative assessment of ^18^F-FDG-PET/CT after exclusion of significant confounders produced cutoff values with good diagnostic accuracy.^6^ Our results did not corroborate these findings in TAVI-IE (Table 3). Comparing our results to the earlier study, ^18^F-FDG-PET/CT seems more likely to underdiagnose TAVI-IE than PVE in general, although we must be cautious in generalizing our findings. Our study contained 5 patients with a false negative ^18^F-FDG-PET/CT scan, who had signs of a vegetation on either CTA (2/5), TTE/TEE (1/5), or both (1/5). This underlines the value of anatomic imaging with CTA and echocardiography (on top of metabolic imaging) in order to detect vegetations which may easily be missed by ^18^F-FDG-PET/CT due to the low inflammatory response associated with vegetations.

**Figure 5:**
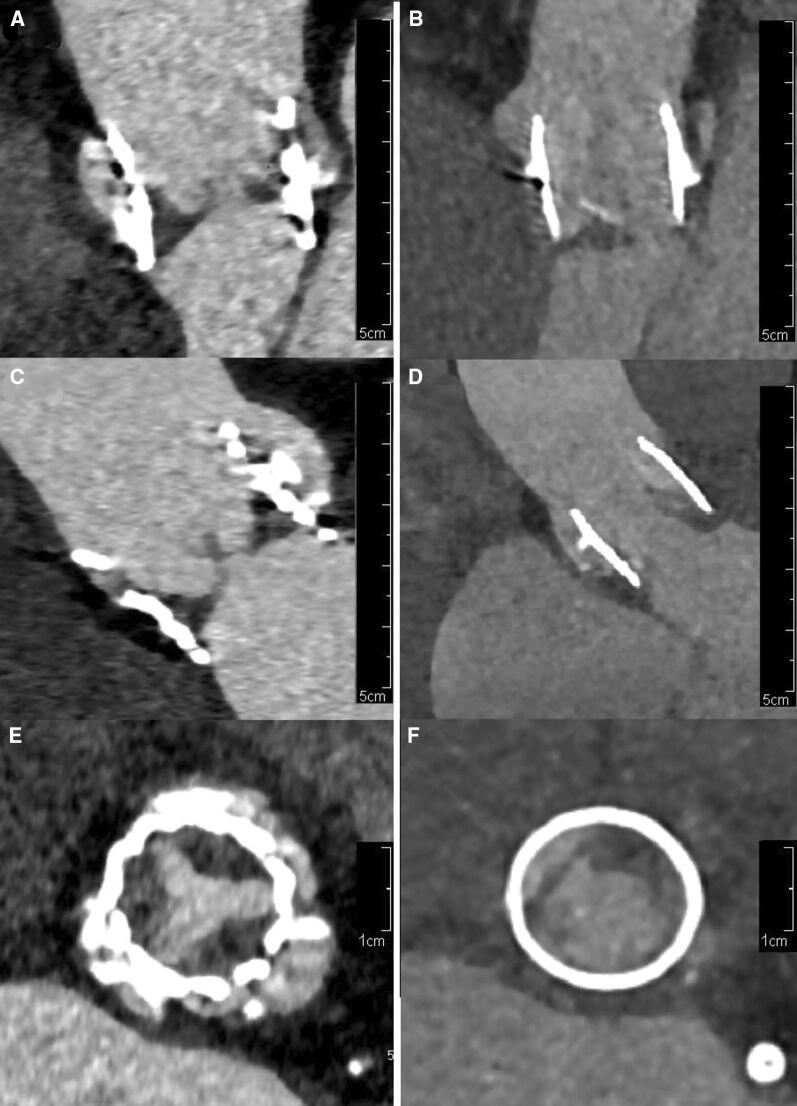
CTA images of a Sapiens valve with signs of leaflet thrombosis (**A**, **C**, **E**) and a Lotus valve with signs of vegetation (**B**, **D**, **F**)

It is important to be aware of potential pitfalls when interpreting valvular abnormalities on CTA. Prosthetic stent material-induced artifacts may obscure valvular abnormality and cause false negative results. On the other hand, leaflet thrombosis (hypo attenuating leaflet thickening, HALT) can occur after TAVI even when patients use anticoagulation therapy.^15^,^16^ HALT may potentially be misinterpreted as a vegetation and can lead to false positive CTA findings. Besides the clinical context, HALT tends to be located at the base of the leaflets and taper toward the free edge, whereas vegetations have a more irregular shape and can be much larger (Figure 5).

The value of ^18^F-FDG-PET/CT and cardiac CTA in the diagnosis of TAVI-IE was, besides case reports,^13^ only shown once before in a recently published case series of 16 patients.^9^ It showed significant potential of this multi-imaging approach and suggested the use of ESC criteria for the diagnosis of TAVI-IE. Our results confirm these findings. Moreover our study demonstrates the additional diagnostic value of ^18^F-FDG-PET/CT and CTA for patients suspected for TAVI-IE. It results in a change of the final diagnosis when the ESC criteria are applied instead of the modified Duke criteria alone and supports a more widened use of these relatively new techniques.

All the mentioned imaging techniques seem to have additional diagnostic value. Although the newer imaging techniques are expensive and associated with some radiation, they provide important extra information allowing a better diagnostic process, which is crucial for these seriously ill patients.

There are several limitations to our study. The most important is the way the final diagnosis was established. Since no patient had undergone surgery, we relied on the ESC criteria and the decision of the Endocarditis Team for the final diagnosis. Since ^18^F-FDG-PET/CT and CTA results were taken into account when making the decision for the final diagnosis, this can be seen as an incorporation bias and thus as a major limitation of this study. However, due to the retrospective design of the study, this could not readily have been prevented. This problem exists in most endocarditis studies as the pathological Duke criteria are often not available.^3^ Additionally, the retrospective nature of the study and relatively small number of patients limit the generalization of our findings to all patients with TAVI-IE.

In conclusion, the addition of ^18^F-FDG-PET/CT and CTA in the work-up of patients with suspected TAVI-IE provided valuable complementary information to echocardiography, resulting in reclassification of 33% of patients in our study.

## New Knowledge Gained

^18^F-FDG-PET/CT and CTA help clinicians to assess patients with TAVI-IE and both of these imaging tools should be considered in the diagnostic work-up of patients with suspected TAVI-IE.

## Electronic supplementary material

Below is the link to the electronic supplementary material.

Electronic supplementary material 1 (DOCX 27 kb)

Electronic supplementary material 2 (PPTX 270 kb)

Electronic supplementary material 3 (M4A 4566 kb)

## Abbreviations

EARL: European Association of Nuclear Medicine Research Ltd
ESC: European Society of Cardiology
PET: Positron emission tomography
PVE: Prosthetic heart valve endocarditis
SUV: Standardized uptake value
TAV: Transcatheter aortic valve
TAVI-IE: Transcatheter aortic valve infectious endocarditis
TEE: Transesophageal echocardiography
TTE: Transthoracic echocardiogram
^18^F-FDG: ^18^F-fluorodeoxyglucose
