# Supplementary material for: Added value of 18F-FDG-PET/CT and cardiac CTA in suspected transcatheter aortic valve endocarditis
Source: J Nucl Cardiol. 2019 Dec 2;28(5):2072–82. doi: 10.1007/s12350-019-01963-x (PMC8648682; doi:10.1007/s12350-019-01963-x)
Supplement: Supplementary file 1 — Electronic supplementary material 1 (DOCX 27 kb) [file 12350_2019_1963_MOESM1_ESM.docx]

**SUPPLEMNTAL MATERIAL**

**Supplemental table**

**Online Resource 1.** Blood cultures, echocardiographic, PET/CT and CTA findings of patients with suspected TAV-IE along with the different diagnosis based on the modified Duke criteria (without the use of PET/CT and CTA), the modified ESC-criteria and the consensus of the Endocarditis Team.

| **Patient** | **Age**  **(Years)** | **Sex**  **(M/F)** | **Positive blood-cultures (yes/no)** | **Specimen** | **TTE/TEE Findings** | **PET/CT Signs of TAV-IE (+/-)†** | **Days from TAVI implantaion** | **CTA**  **(Findings)** | **Modified Duke Criteria (Major=M, Minor=1)(Rejected TAV-IE=R, Possible TAV-IE= P, Definite TAV-IE= D)** | **ESC Criteria (Rejected TAV-IE=R, Possible TAV-IE= P, Definite TAV-IE= D)** | **Additional findings on PET/CT** | **Final diagnosis**  **based on Endocarditis Team consensus)** | **Mortality during follow up** | **Follow up duration**  **(days)** |
| --- | --- | --- | --- | --- | --- | --- | --- | --- | --- | --- | --- | --- | --- | --- |
| **1** | 75 | F | Yes | *S. aureus* | Non-diagnostic* | + | 126 | Myc. Aneurysm | 1M, 2m (P) | 2M, 2m(D) | None | Definite TAV-IE | No | 1127 |
| **2** | 81 | F | Yes | *S. oralis* | Vegetation | + | 557 | Vegetation | 2M, 3m (D) | 2M, 3m (D) | Spondylidiscitis, Bursitis trochanterica right | Definite TAV-IE | Yes | 66 |
| **3** | 77 | M | Yes | *E. faecalis* | Vegetation | + | 437 | Vegetation and Myc. Aneurysm | 2M, 3m (D) | 2M, 3m (D) | Splenic abscess | Definite TAV-IE | No | 1144 |
| **4** | 65 | M | Yes | *S. epidermidis* | Normal | + | 76 | NA | 1M, 2m (P) | 2M, 2m (D) | Pneumonia | Definite TAV-IE | Yes | 1037 |
| **5** | 71 | F | Yes | *E. faecalis* | Vegetation | + | 106 | Normal | 2M, 2m (D) | 2M, 2m (D) | Possible erysipelas/ reactive inflammation both lower legs | Definite TAVI Endocarditis | No | 1023 |
| **6** | 79 | M | Yes | *S. aureus* | Normal | - | 105 | Vegetation | 1M, 2m (P) | 2M, 2m (D) | Synovitis left knee | Definite TAV-IE | Yes | 135 |
| **7** | 84 | M | Yes | *E. faecalis* | Normal | - | 122 | Normal | 1M, 1m (P) | 1M, 1m (P) | FDG uptake around PM lead, pleural plaques, active mediastinal lymph nodes, abnormal left adrenal gland | PM-lead infection | Yes | 465 |
| **8** | 68 | M | Yes | *S. lugdunensis* | Normal | + | 18 | NA | 0M, 3m (P) | 1M, 3m (D) | Sigmoid carcinoma, path. inguinal lymphnodes, active medistinal lymphnodes | Definite TAV-IE | Yes | 496 |
| **9** | 68 | M | Yes | *M. abcessus.* | Normal | - | 128 | NA | 0M, 2m (R) | 0M, 2m (R) | Pneumonia with pleural empyema | Pleural Empyema | Yes | 117 |
| **10** | 72 | F | Yes | *S. oralis* | Non-diagnostic* | - | 46 | Normal | 1M, 3m (D) | 1M, 3m (D) | Pneumonia | Definite TAV-IE‡ | Yes | 113 |
| **11** | 89 | F | Yes | *S. bovis* | Normal | - | 1908 | NA | 1M, 2m (P) | 1M, 2m (P) | Coloncarcinoma, mitral valve endocarditis | Possible TAV-IE /MV endocarditis | No | 850 |
| **12** | 70 | F | Yes | *S. epidermidis* | Normal | - | 54 | Normal | 1M, 1m (P) | 1M, 1m (P) | PM pocket infection, reactive bone marrow, adrenal hyperplasia | Central line infection | Yes | 105 |
| **13** | 87 | F | Yes | *E. faecalis* | Normal | - | 162 | Normal | 1M, 2m (P) | 1M, 2m (P) | NA | Rejected TAV-IE | No | 128 |
| **14** | 81 | F | Yes | *S. sanguinis* | Vegetation | - | 835 | Vegetation | 2M, 2m (D) | 2M, 2m (D) | None | Definite TAV-IE | Yes | 29 |
| **15** | 71 | M | Yes | *E. faecalis* | Non-diagnostic* | - | 1362 | NA | 1M, 2m (P) | 1M, 2m (P) | Skeletal FDG uptake left ventrolateral costae | Infected Hematoma | Yes | 50 |
| **16** | 81 | M | Yes | *E. faecalis* | Normal | - | 692 | NA | 1M, 2m (P) | 1M, 2m (P) | NA | Possible TAV-IE | No | 896 |
| **17** | 86 | M | Yes | *S. mitis* | Normal | - | 668 | NA | 1M, 2m (P) | 1M, 2m (P) | Abnormal FDG uptake in the prostate, left femur and prosthetic hip | Possible TAVI Endocarditis | No | 915 |
| **18** | 94 | M | Yes | *S. lugdunensis* | Normal | - | 121 | NA | 1M, 2m (P) | 1M, 2m (P) | None | Spondylodiscitis | Yes | 181 |
| **19** | 78 | F | Yes | *S. mitis* | Normal | - | 1212 | NA | 1M, 1m (P) | 1M, 1m (P) | None | Possible TAV-IE | No | 736 |
| **20** | 89 | M | Yes | *E. faecalis* | Normal | - | 1768 | NA | 1M, 1m (P) | 1M, 1m (P) | Abnormal FDG uptake in the left prosthetic knee and flexura hepatica | Possible TAV-IE | No | 245 |
| **21** | 91 | F | No | *NA* | Vegetation | - | 448 | NA | 1M, 1m (P) | 1M, 1m (P) | Possibly infected native mitral valve | Possible TAV-IE | No | 3096 |
| **22** | 77 | M | Yes | *E. faecalis* | Normal | - | 36 | NA | 1M, 2m (P) | 1M, 2m (P) | Abnormal FDG uptake in the hilar lymph nodes | Possible TAV-IE | No | 2420 |
| **23** | 84 | M | Yes | *E. faecalis* | Vegetation | - | 839 | NA | 2M, 2m (D) | 2M, 2m (D) | None | Definite TAV-IE | No | 1339 |
| **24** | 68 | M | Yes | *E. faecalis* | Normal | - | 55 | Vegetation | 1M, 2m (P) | 2M, 2m (D) | Abnormality in the urethra | Definite TAV-IE | No | 1309 |
| **25** | 73 | M | Yes | *S. mitis* | Normal | - | 393 | NA | 1M, 2m (P) | 1M, 2m (P) | Diffuse bone marrow activity | Possible TAV-IE | No | 602 |
| **26** | 52 | F | No | *NA* | Vegetation | + | 746 | Vegetation | 1M, 3m (P) | 1M, 3m (P) | Costosternal fractures, abnormal splenic FDG uptake | Definite TAV-IE‡ | No | 171 |
| **27** | 88 | F | Yes | *S. infantarius* | Normal | - | 100 | NA | 0M,3m (P) | 0M, 3m (P) | None | Possible TAV-IE | Yes | 83 |
| **28** | 80 | M | Yes | *L. rhamnosus* | New paravalvular leak | - | 603 | NA | 1M, 2m (P) | 1M,2m (P) | None | Possible TAV-IE | Yes | 28 |
| **29** | 71 | F | Yes | *E. faecalis* | Normal | - | 64 | NA | 0M, 3 (P) | 0M, 3m (P) | None | Possible TAV-IE | Yes | 1213 |
| **30** | 49 | M | Yes | *E. faecalis* | Normal | - | 1531 | Normal | 1M, 2m (P) | 1M,2m (P) | None | Possible TAV-IE | No | 117 |

* Uncertain findings on the TTE and/or TEE were signified as non-diagnostic.

† Positive signs of TAVI endocarditis are indicated as a “+” and negative signs of TAVI endocarditis are indicated as “- “.

‡ Patient had 1 major (positive blood cultures or positive imaging) and 3 minor (Fever, prosthetic valve, signs of embolization) criteria for endocarditis
