# Supplementary material for: Added value of 18F-FDG-PET/CT and cardiac CTA in suspected transcatheter aortic valve endocarditis
Source: J Nucl Cardiol. 2019 Dec 2;28(5):2072–82. doi: 10.1007/s12350-019-01963-x (PMC8648682; doi:10.1007/s12350-019-01963-x)
Supplement: Supplementary file 2 — Electronic supplementary material 2 (PPTX 270 kb) [file 12350_2019_1963_MOESM2_ESM.pptx]

## Slide 1
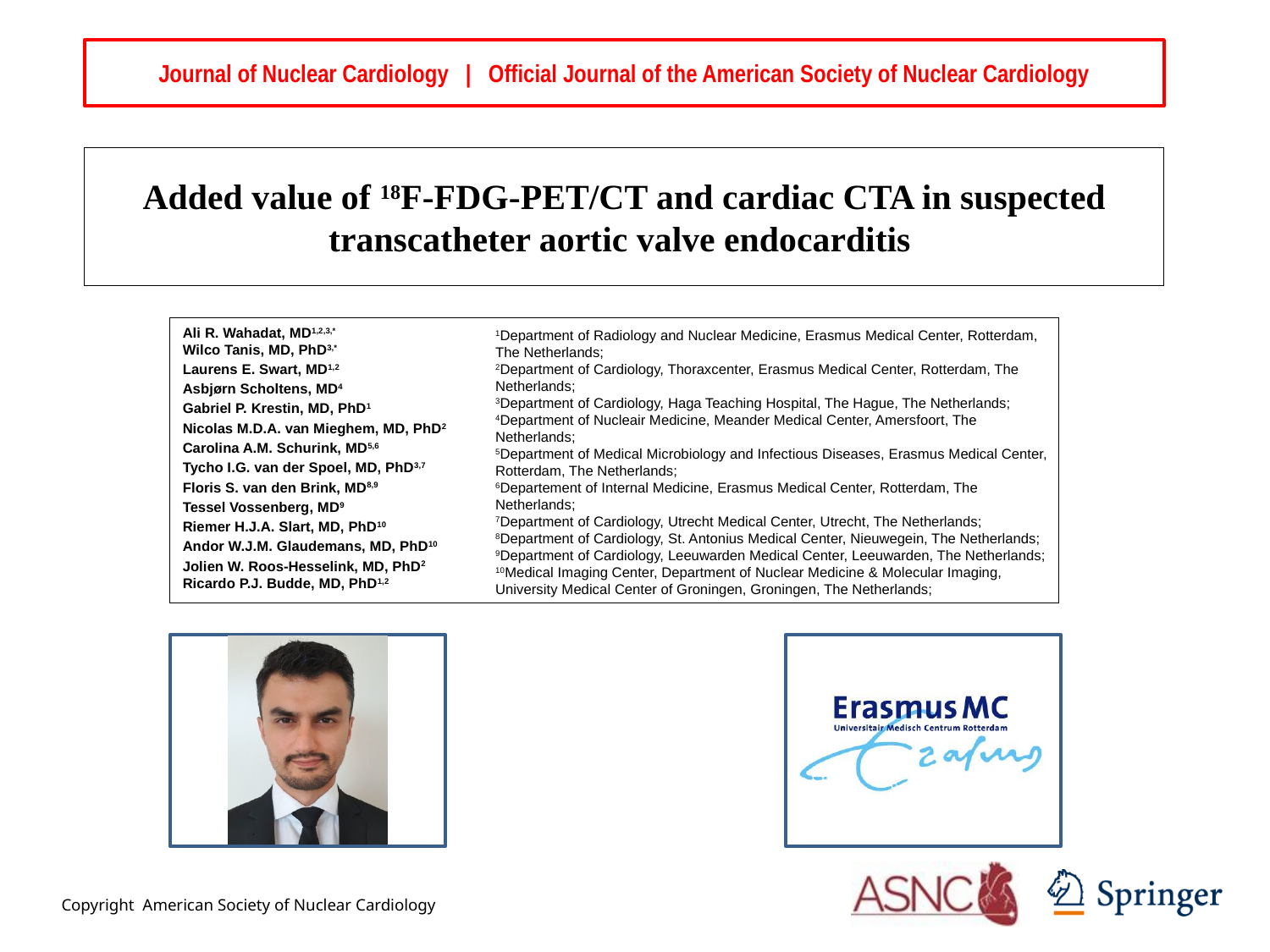

Journal of Nuclear Cardiology | Official Journal of the American Society of Nuclear Cardiology
# Added value of 18F-FDG-PET/CT and cardiac CTA in suspected transcatheter aortic valve endocarditis
Ali R. Wahadat, MD1,2,3,*
Wilco Tanis, MD, PhD3,*
Laurens E. Swart, MD1,2
Asbjørn Scholtens, MD4
Gabriel P. Krestin, MD, PhD1
Nicolas M.D.A. van Mieghem, MD, PhD2
Carolina A.M. Schurink, MD5,6
Tycho I.G. van der Spoel, MD, PhD3,7
Floris S. van den Brink, MD8,9
Tessel Vossenberg, MD9
Riemer H.J.A. Slart, MD, PhD10
Andor W.J.M. Glaudemans, MD, PhD10
Jolien W. Roos-Hesselink, MD, PhD2
Ricardo P.J. Budde, MD, PhD1,2
1Department of Radiology and Nuclear Medicine, Erasmus Medical Center, Rotterdam, The Netherlands;
2Department of Cardiology, Thoraxcenter, Erasmus Medical Center, Rotterdam, The Netherlands;
3Department of Cardiology, Haga Teaching Hospital, The Hague, The Netherlands;
4Department of Nucleair Medicine, Meander Medical Center, Amersfoort, The Netherlands;
5Department of Medical Microbiology and Infectious Diseases, Erasmus Medical Center, Rotterdam, The Netherlands;
6Departement of Internal Medicine, Erasmus Medical Center, Rotterdam, The Netherlands;
7Department of Cardiology, Utrecht Medical Center, Utrecht, The Netherlands;
8Department of Cardiology, St. Antonius Medical Center, Nieuwegein, The Netherlands;
9Department of Cardiology, Leeuwarden Medical Center, Leeuwarden, The Netherlands;
10Medical Imaging Center, Department of Nuclear Medicine & Molecular Imaging, University Medical Center of Groningen, Groningen, The Netherlands;
Copyright American Society of Nuclear Cardiology

## Slide 2
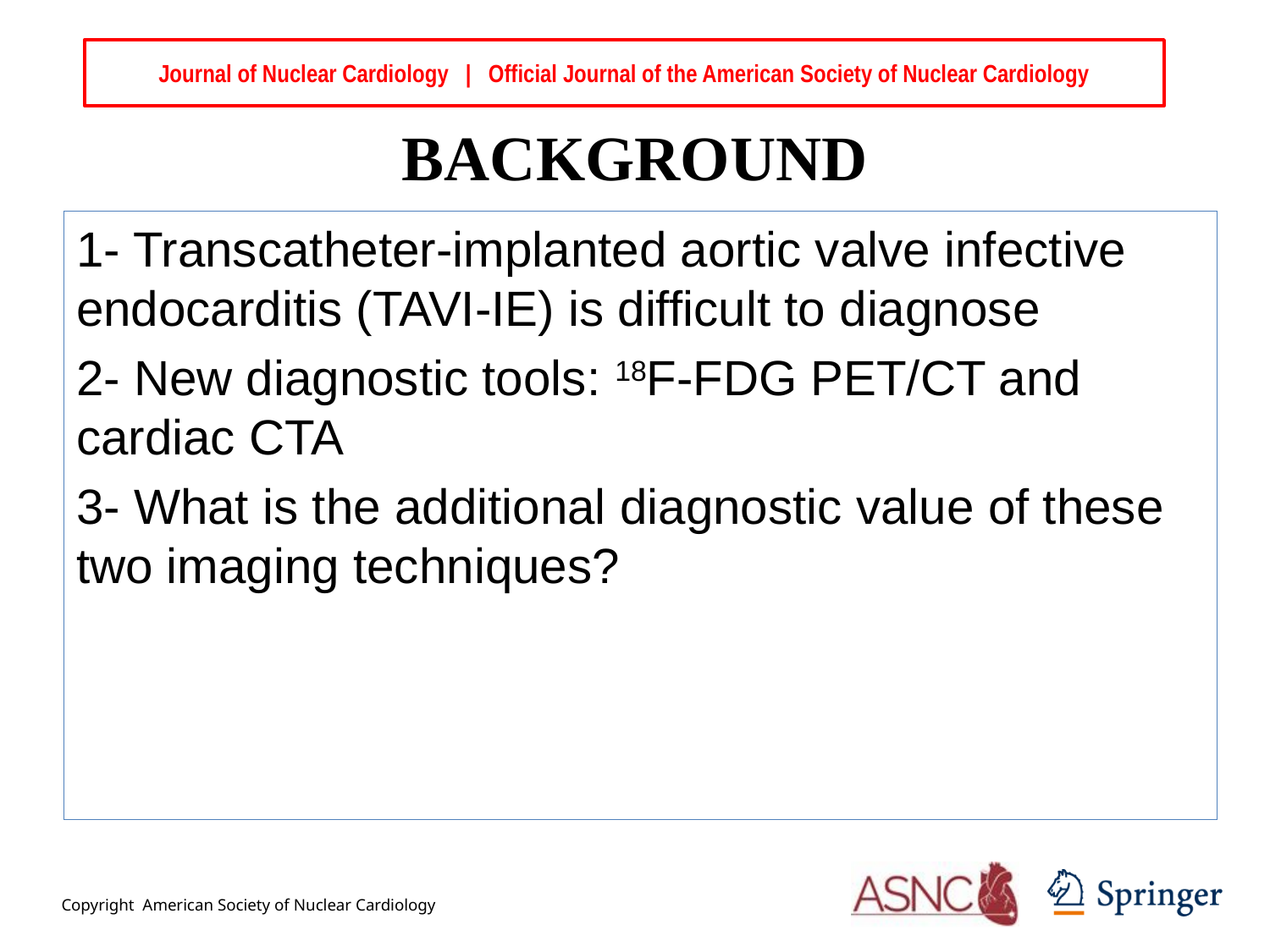

Journal of Nuclear Cardiology | Official Journal of the American Society of Nuclear Cardiology
# BACKGROUND
1- Transcatheter-implanted aortic valve infective endocarditis (TAVI-IE) is difficult to diagnose
2- New diagnostic tools: 18F-FDG PET/CT and cardiac CTA
3- What is the additional diagnostic value of these two imaging techniques?
Copyright American Society of Nuclear Cardiology

## Slide 3
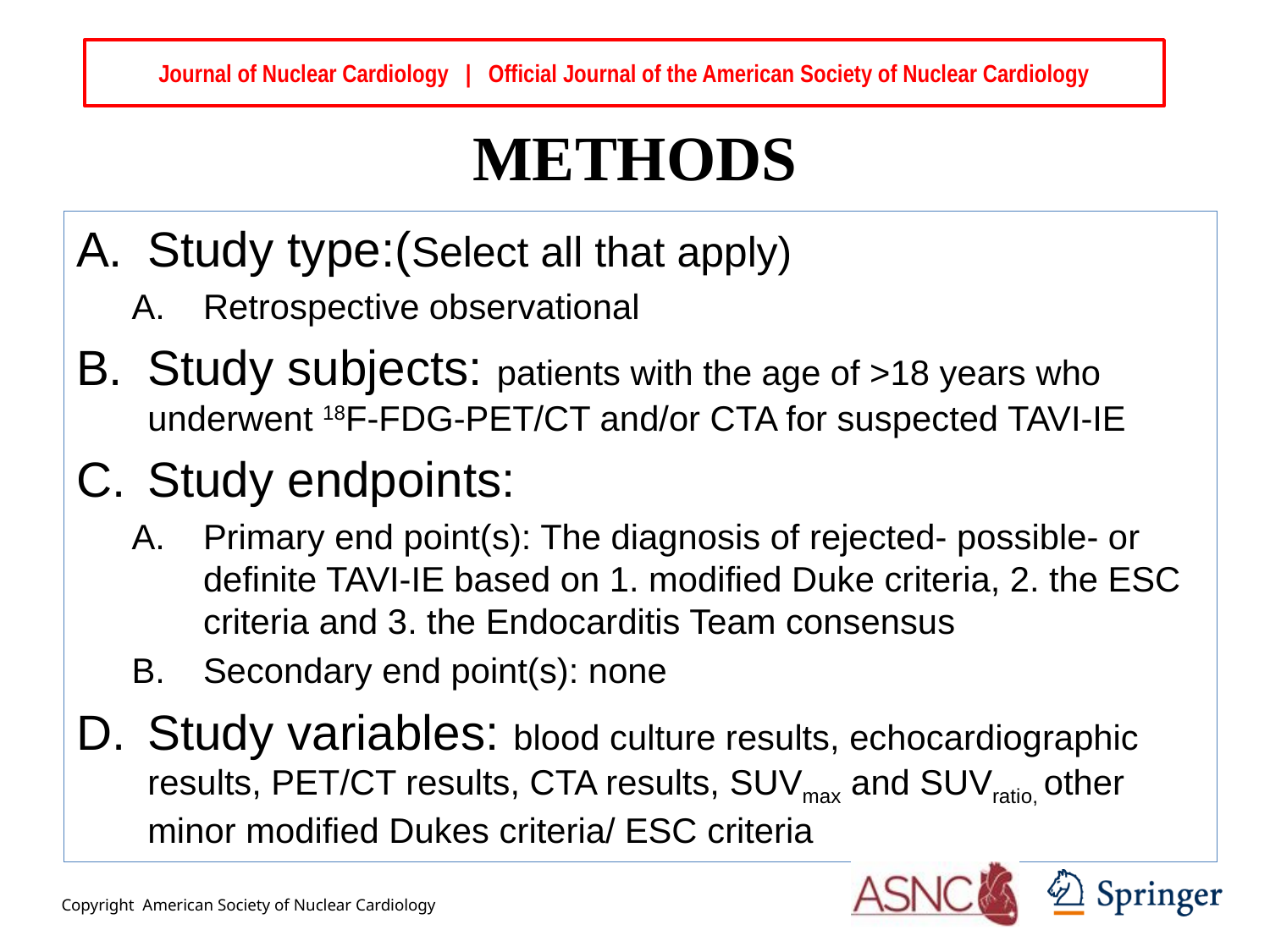

Journal of Nuclear Cardiology | Official Journal of the American Society of Nuclear Cardiology
# METHODS
Study type:(Select all that apply)
Retrospective observational
Study subjects: patients with the age of >18 years who underwent 18F-FDG-PET/CT and/or CTA for suspected TAVI-IE
Study endpoints:
Primary end point(s): The diagnosis of rejected- possible- or definite TAVI-IE based on 1. modified Duke criteria, 2. the ESC criteria and 3. the Endocarditis Team consensus
Secondary end point(s): none
Study variables: blood culture results, echocardiographic results, PET/CT results, CTA results, SUVmax and SUVratio, other minor modified Dukes criteria/ ESC criteria
Copyright American Society of Nuclear Cardiology

## Slide 4
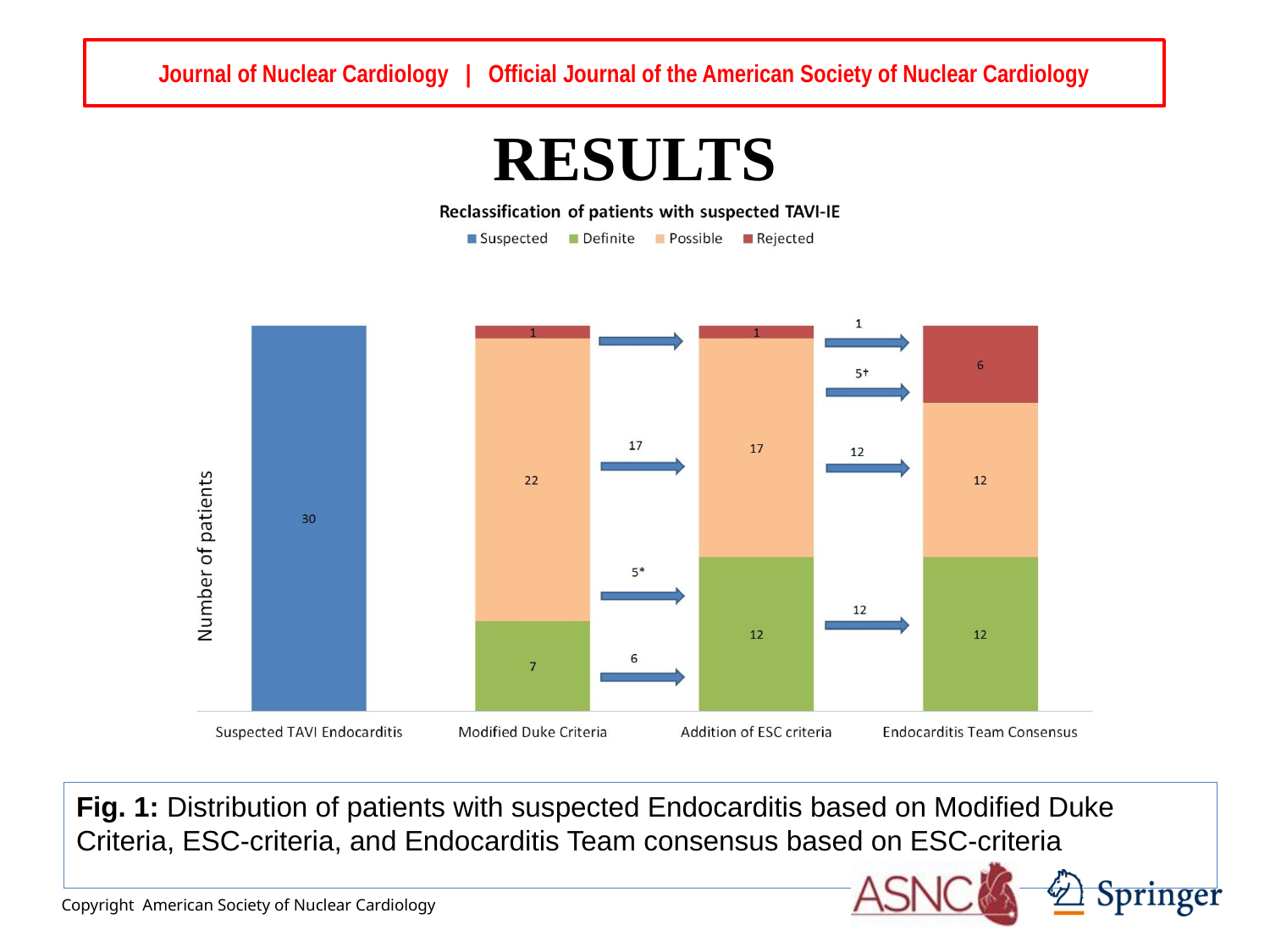

Journal of Nuclear Cardiology | Official Journal of the American Society of Nuclear Cardiology
# RESULTS
Fig. 1: Distribution of patients with suspected Endocarditis based on Modified Duke Criteria, ESC-criteria, and Endocarditis Team consensus based on ESC-criteria
Copyright American Society of Nuclear Cardiology

## Slide 5
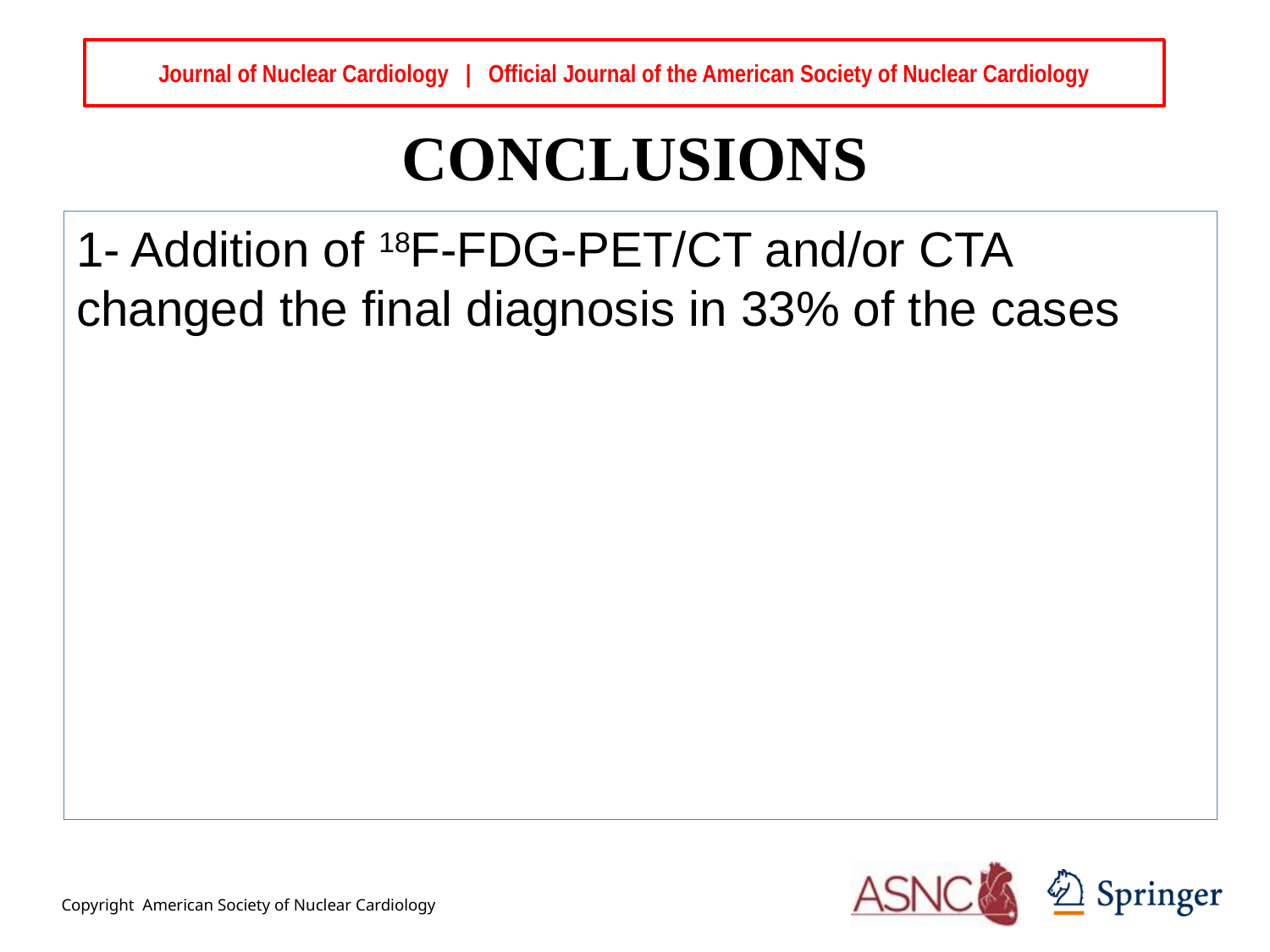

Journal of Nuclear Cardiology | Official Journal of the American Society of Nuclear Cardiology
# CONCLUSIONS
1- Addition of 18F-FDG-PET/CT and/or CTA changed the final diagnosis in 33% of the cases
Copyright American Society of Nuclear Cardiology
